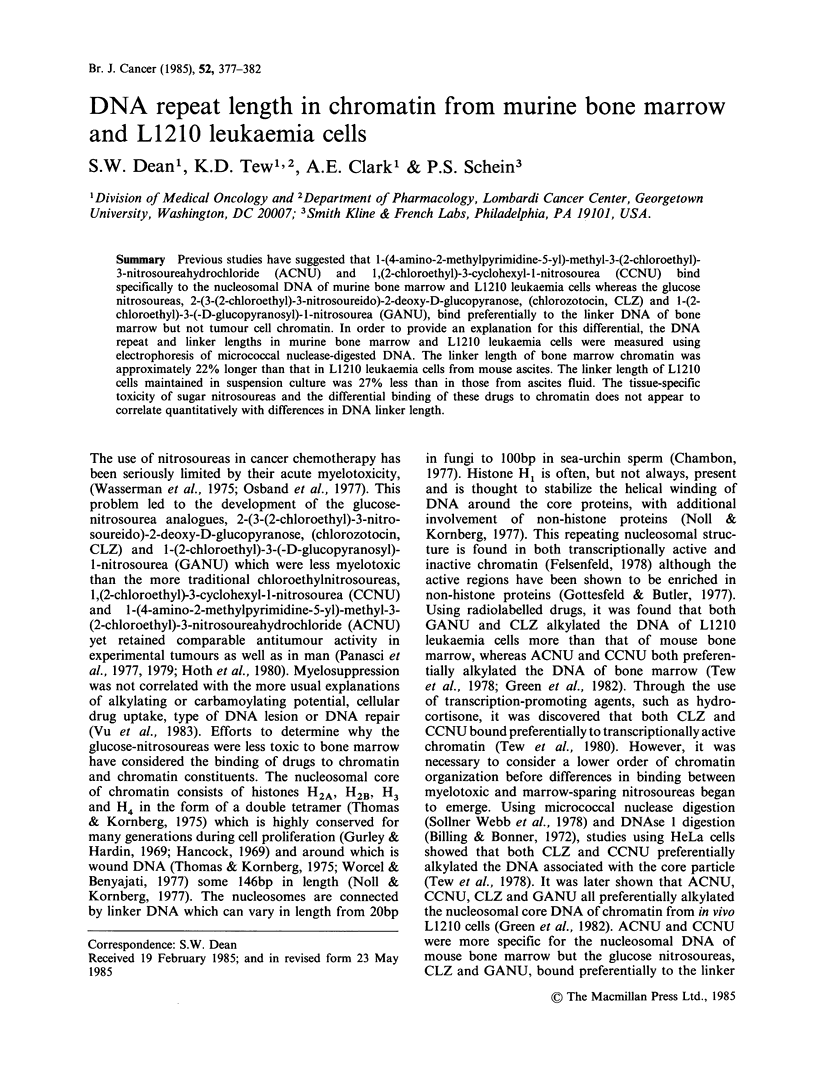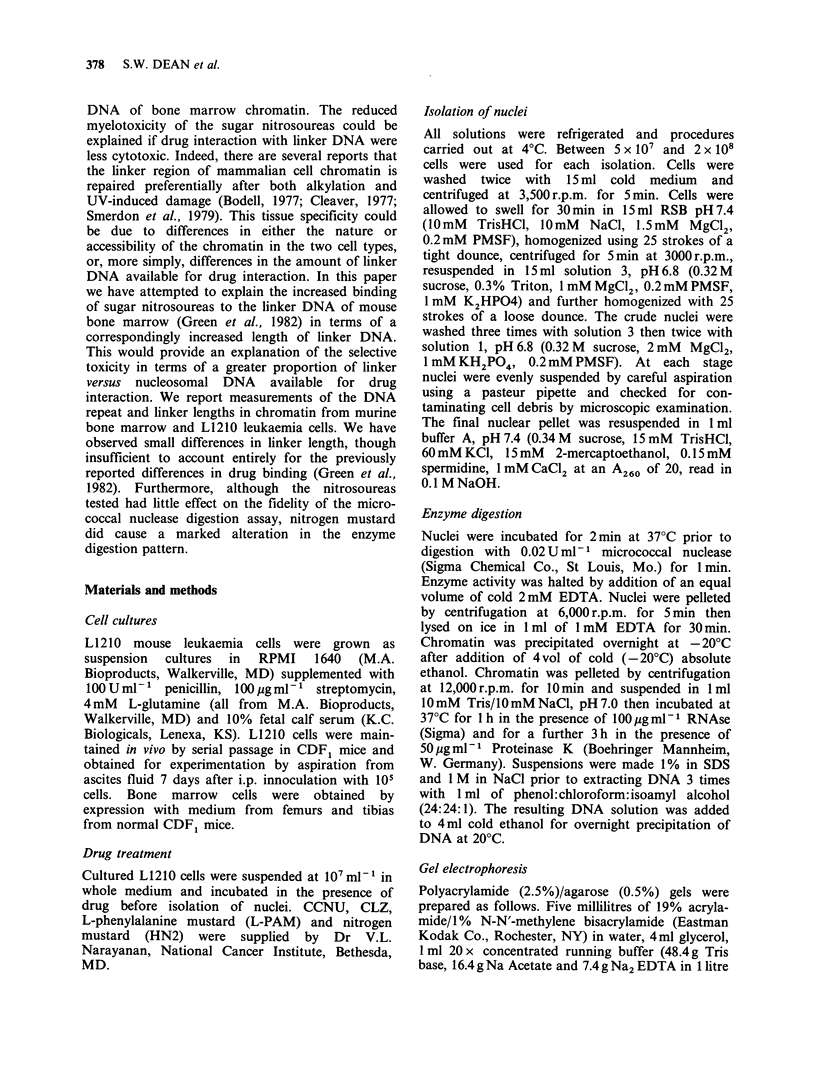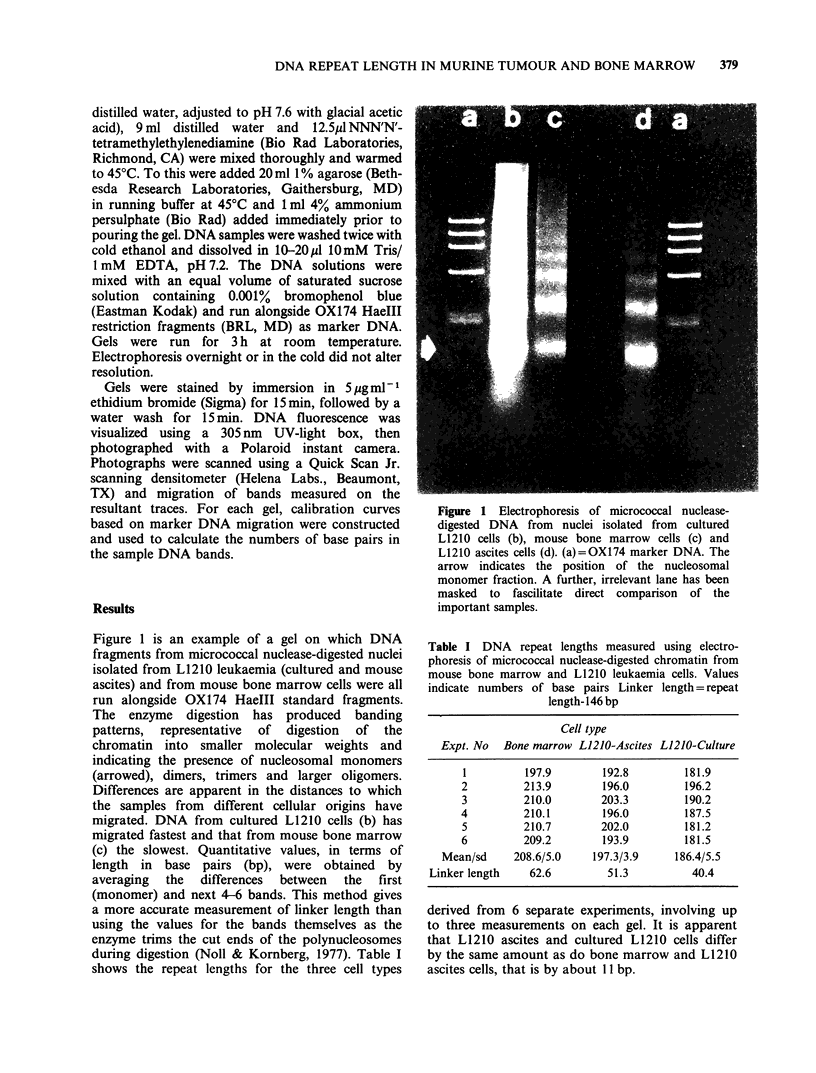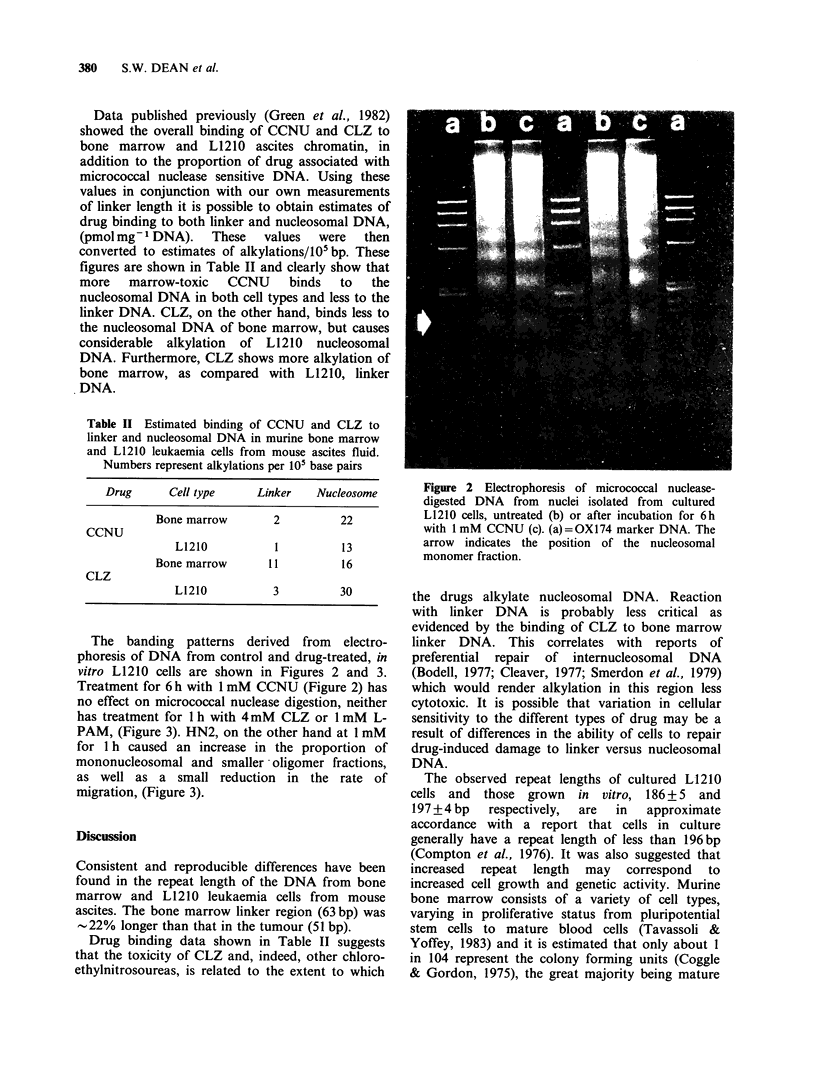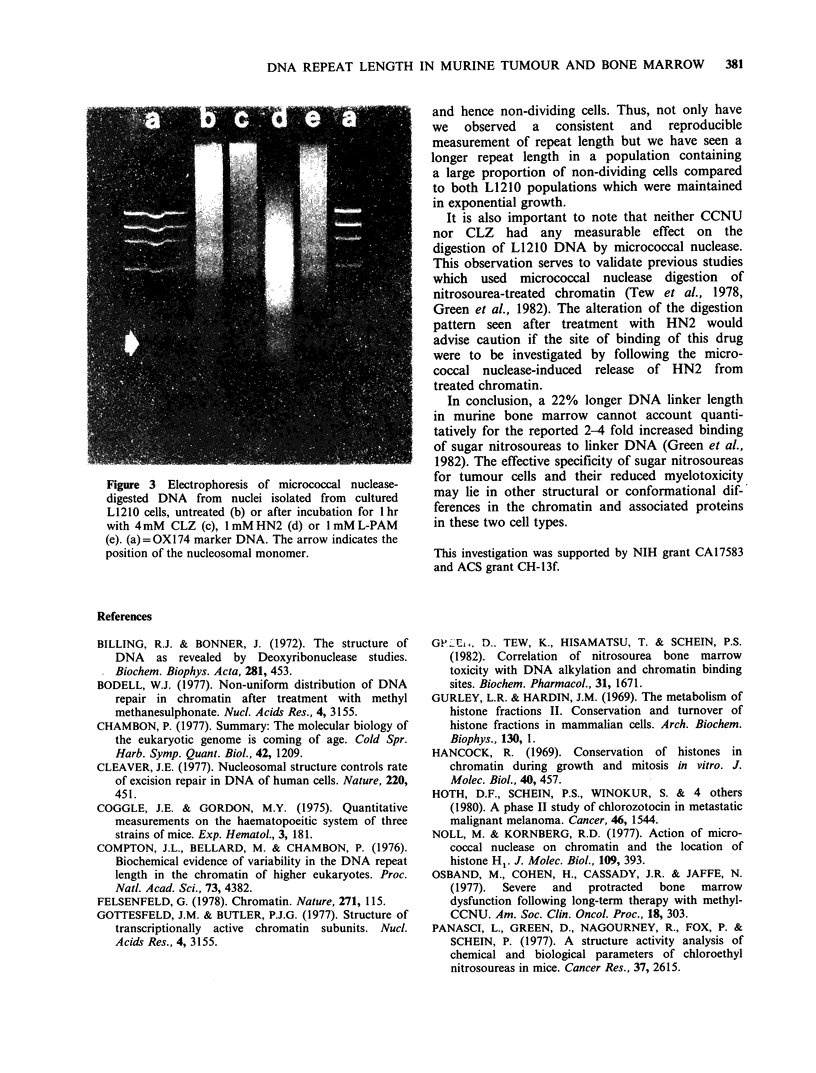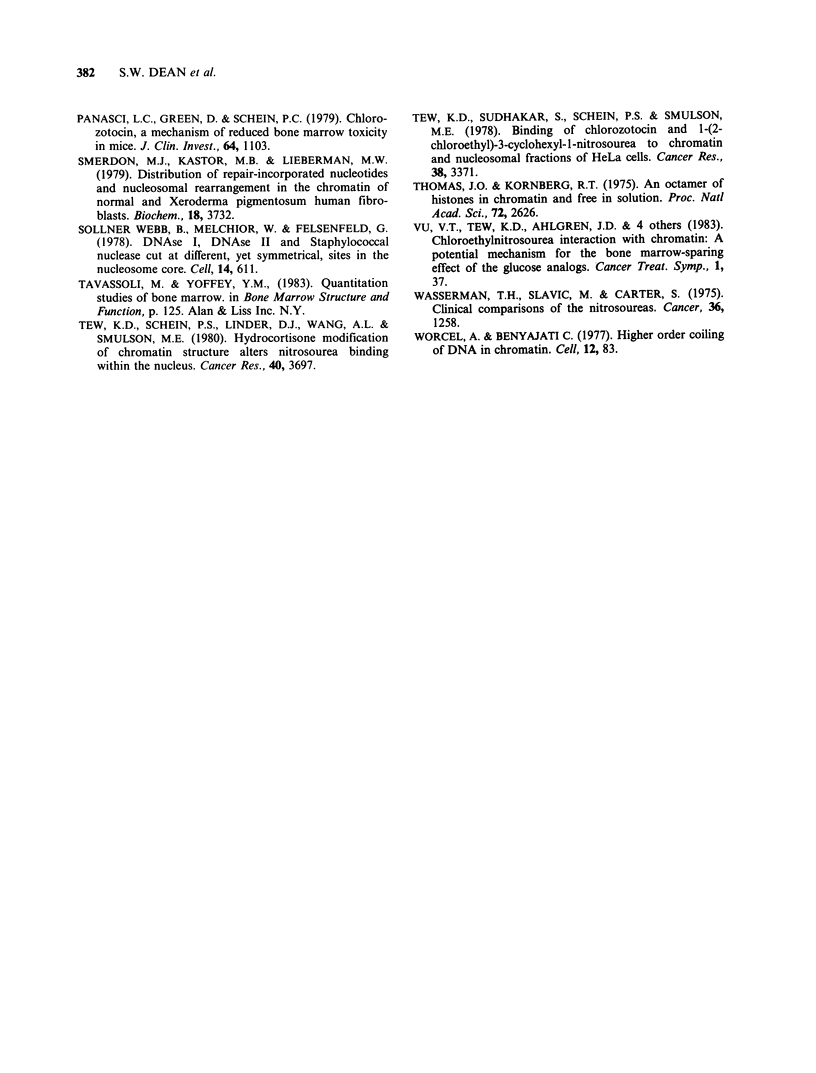# DNA repeat length in chromatin from murine bone marrow and L1210 leukaemia cells.

**DOI:** 10.1038/bjc.1985.204

**Published:** 1985-09

**Authors:** S. W. Dean, K. D. Tew, A. E. Clark, P. S. Schein

## Abstract

**Images:**


Br. J. Cancer (1985), **52**, 377–382

### DNA repeat length in chromatin from murine bone marrow and L1210 leukaemia cells

S.W. Dean[1], K.D. Tew[1,2], A.E. Clark[1] & P.S. Schein[3]

[1]*Division of Medical Oncology and* [2]*Department of Pharmacology, Lombardi Cancer Center, Georgetown University, Washington, DC 20007;* [3]*Smith Kline & French Labs, Philadelphia, PA 19101, USA.*

**Summary** Previous studies have suggested that 1-(4-amino-2-methylpyrimidine-5-yl)-methyl-3-(2-chloroethyl)-3-nitrosoureahydrochloride (ACNU) and 1,(2-chloroethyl)-3-cyclohexyl-1-nitrosourea (CCNU) bind specifically to the nucleosomal DNA of murine bone marrow and L1210 leukaemia cells whereas the glucose nitrosoureas, 2-(3-(2-chloroethyl)-3-nitrosoureido)-2-deoxy-D-glucopyranose, (chlorozotocin, CLZ) and 1-(2-chloroethyl)-3-(-D-glucopyranosyl)-1-nitrosourea (GANU), bind preferentially to the linker DNA of bone marrow but not tumour cell chromatin. In order to provide an explanation for this differential, the DNA repeat and linker lengths in murine bone marrow and L1210 leukaemia cells were measured using electrophoresis of micrococcal nuclease-digested DNA. The linker length of bone marrow chromatin was approximately 22% longer than that in L1210 leukaemia cells from mouse ascites. The linker length of L1210 cells maintained in suspension culture was 27% less than in those from ascites fluid. The tissue-specific toxicity of sugar nitrosoureas and the differential binding of these drugs to chromatin does not appear to correlate quantitatively with differences in DNA linker length.

The use of nitrosoureas in cancer chemotherapy has been seriously limited by their acute myelotoxicity, (Wasserman *et al.*, 1975; Osband *et al.*, 1977). This problem led to the development of the glucose-nitrosourea analogues, 2-(3-(2-chloroethyl)-3-nitrosoureido)-2-deoxy-D-glucopyranose, (chlorozotocin, CLZ) and 1-(2-chloroethyl)-3-(-D-glucopyranosyl)-1-nitrosourea (GANU) which were less myelotoxic than the more traditional chloroethylnitrosoureas, 1,(2-chloroethyl)-3-cyclohexyl-1-nitrosourea (CCNU) and 1-(4-amino-2-methylpyrimidine-5-yl)-methyl-3-(2-chloroethyl)-3-nitrosoureahydrochloride (ACNU) yet retained comparable antitumour activity in experimental tumours as well as in man (Panasci *et al.*, 1977, 1979; Hoth *et al.*, 1980). Myelosuppression was not correlated with the more usual explanations of alkylating or carbamoylating potential, cellular drug uptake, type of DNA lesion or DNA repair (Vu *et al.*, 1983). Efforts to determine why the glucose-nitrosoureas were less toxic to bone marrow have considered the binding of drugs to chromatin and chromatin constituents. The nucleosomal core of chromatin consists of histones $H_{2A}$, $H_{2B}$, $H_3$ and $H_4$ in the form of a double tetramer (Thomas & Kornberg, 1975) which is highly conserved for many generations during cell proliferation (Gurley & Hardin, 1969; Hancock, 1969) and around which is wound DNA (Thomas & Kornberg, 1975; Worcel & Benyajati, 1977) some 146bp in length (Noll & Kornberg, 1977). The nucleosomes are connected by linker DNA which can vary in length from 20bp in fungi to 100bp in sea-urchin sperm (Chambon, 1977). Histone $H_1$ is often, but not always, present and is thought to stabilize the helical winding of DNA around the core proteins, with additional involvement of non-histone proteins (Noll & Kornberg, 1977). This repeating nucleosomal structure is found in both transcriptionally active and inactive chromatin (Felsenfeld, 1978) although the active regions have been shown to be enriched in non-histone proteins (Gottesfeld & Butler, 1977). Using radiolabelled drugs, it was found that both GANU and CLZ alkylated the DNA of L1210 leukaemia cells more than that of mouse bone marrow, whereas ACNU and CCNU both preferentially alkylated the DNA of bone marrow (Tew *et al.*, 1978; Green *et al.*, 1982). Through the use of transcription-promoting agents, such as hydrocortisone, it was discovered that both CLZ and CCNU bound preferentially to transcriptionally active chromatin (Tew *et al.*, 1980). However, it was necessary to consider a lower order of chromatin organization before differences in binding between myelotoxic and marrow-sparing nitrosoureas began to emerge. Using micrococcal nuclease digestion (Sollner Webb *et al.*, 1978) and DNAse 1 digestion (Billing & Bonner, 1972), studies using HeLa cells showed that both CLZ and CCNU preferentially alkylated the DNA associated with the core particle (Tew *et al.*, 1978). It was later shown that ACNU, CCNU, CLZ and GANU all preferentially alkylated the nucleosomal core DNA of chromatin from *in vivo* L1210 cells (Green *et al.*, 1982). ACNU and CCNU were more specific for the nucleosomal DNA of mouse bone marrow but the glucose nitrosoureas, CLZ and GANU, bound preferentially to the linker

Correspondence: S.W. Dean
Received 19 February 1985; and in revised form 23 May 1985

© The Macmillan Press Ltd., 1985

DNA of bone marrow chromatin. The reduced myelotoxicity of the sugar nitrosoureas could be explained if drug interaction with linker DNA were less cytotoxic. Indeed, there are several reports that the linker region of mammalian cell chromatin is repaired preferentially after both alkylation and UV-induced damage (Bodell, 1977; Cleaver, 1977; Smerdon *et al.*, 1979). This tissue specificity could be due to differences in either the nature or accessibility of the chromatin in the two cell types, or, more simply, differences in the amount of linker DNA available for drug interaction. In this paper we have attempted to explain the increased binding of sugar nitrosoureas to the linker DNA of mouse bone marrow (Green *et al.*, 1982) in terms of a correspondingly increased length of linker DNA. This would provide an explanation of the selective toxicity in terms of a greater proportion of linker *versus* nucleosomal DNA available for drug interaction. We report measurements of the DNA repeat and linker lengths in chromatin from murine bone marrow and L1210 leukaemia cells. We have observed small differences in linker length, though insufficient to account entirely for the previously reported differences in drug binding (Green *et al.*, 1982). Furthermore, although the nitrosoureas tested had little effect on the fidelity of the micrococcal nuclease digestion assay, nitrogen mustard did cause a marked alteration in the enzyme digestion pattern.

### Materials and methods

### *Cell cultures*

L1210 mouse leukaemia cells were grown as suspension cultures in RPMI 1640 (M.A. Bioproducts, Walkerville, MD) supplemented with 100 U ml$^{-1}$ penicillin, 100 $\mu$g ml$^{-1}$ streptomycin, 4 mM L-glutamine (all from M.A. Bioproducts, Walkerville, MD) and 10% fetal calf serum (K.C. Biologicals, Lenexa, KS). L1210 cells were maintained *in vivo* by serial passage in $CDF_1$ mice and obtained for experimentation by aspiration from ascites fluid 7 days after i.p. innoculation with $10^5$ cells. Bone marrow cells were obtained by expression with medium from femurs and tibias from normal $CDF_1$ mice.

### *Drug treatment*

Cultured L1210 cells were suspended at $10^7$ ml$^{-1}$ in whole medium and incubated in the presence of drug before isolation of nuclei. CCNU, CLZ, L-phenylalanine mustard (L-PAM) and nitrogen mustard (HN2) were supplied by Dr V.L. Narayanan, National Cancer Institute, Bethesda, MD.

### *Isolation of nuclei*

All solutions were refrigerated and procedures carried out at 4°C. Between $5 \times 10^7$ and $2 \times 10^8$ cells were used for each isolation. Cells were washed twice with 15 ml cold medium and centrifuged at 3,500 r.p.m. for 5 min. Cells were allowed to swell for 30 min in 15 ml RSB pH 7.4 (10 mM TrisHCl, 10 mM NaCl, 1.5 mM $MgCl_2$, 0.2 mM PMSF), homogenized using 25 strokes of a tight dounce, centrifuged for 5 min at 3000 r.p.m., resuspended in 15 ml solution 3, pH 6.8 (0.32 M sucrose, 0.3% Triton, 1 mM $MgCl_2$, 0.2 mM PMSF, 1 mM $K_2HPO4$) and further homogenized with 25 strokes of a loose dounce. The crude nuclei were washed three times with solution 3 then twice with solution 1, pH 6.8 (0.32 M sucrose, 2 mM $MgCl_2$, 1 mM $KH_2PO_4$, 0.2 mM PMSF). At each stage nuclei were evenly suspended by careful aspiration using a pasteur pipette and checked for contaminating cell debris by microscopic examination. The final nuclear pellet was resuspended in 1 ml buffer A, pH 7.4 (0.34 M sucrose, 15 mM TrisHCl, 60 mM KCl, 15 mM 2-mercaptoethanol, 0.15 mM spermidine, 1 mM $CaCl_2$ at an $A_{260}$ of 20, read in 0.1 M NaOH.

### *Enzyme digestion*

Nuclei were incubated for 2 min at 37°C prior to digestion with 0.02 U ml$^{-1}$ micrococcal nuclease (Sigma Chemical Co., St Louis, Mo.) for 1 min. Enzyme activity was halted by addition of an equal volume of cold 2 mM EDTA. Nuclei were pelleted by centrifugation at 6,000 r.p.m. for 5 min then lysed on ice in 1 ml of 1 mM EDTA for 30 min. Chromatin was precipitated overnight at −20°C after addition of 4 vol of cold (−20°C) absolute ethanol. Chromatin was pelleted by centrifugation at 12,000 r.p.m. for 10 min and suspended in 1 ml 10 mM Tris/10 mM NaCl, pH 7.0 then incubated at 37°C for 1 h in the presence of 100 $\mu$g ml$^{-1}$ RNAse (Sigma) and for a further 3 h in the presence of 50 $\mu$g ml$^{-1}$ Proteinase K (Boehringer Mannheim, W. Germany). Suspensions were made 1% in SDS and 1 M in NaCl prior to extracting DNA 3 times with 1 ml of phenol:chloroform:isoamyl alcohol (24:24:1). The resulting DNA solution was added to 4 ml cold ethanol for overnight precipitation of DNA at 20°C.

### *Gel electrophoresis*

Polyacrylamide (2.5%)/agarose (0.5%) gels were prepared as follows. Five millilitres of 19% acrylamide/1% N-N′-methylene bisacrylamide (Eastman Kodak Co., Rochester, NY) in water, 4 ml glycerol, 1 ml 20× concentrated running buffer (48.4 g Tris base, 16.4 g Na Acetate and 7.4 g $Na_2$ EDTA in 1 litre distilled water, adjusted to pH 7.6 with glacial acetic acid), 9 ml distilled water and 12.5 μl NNN'N'-tetramethylethylenediamine (Bio Rad Laboratories, Richmond, CA) were mixed thoroughly and warmed to 45°C. To this were added 20 ml 1% agarose (Bethesda Research Laboratories, Gaithersburg, MD) in running buffer at 45°C and 1 ml 4% ammonium persulphate (Bio Rad) added immediately prior to pouring the gel. DNA samples were washed twice with cold ethanol and dissolved in 10–20 μl 10 mM Tris/1 mM EDTA, pH 7.2. The DNA solutions were mixed with an equal volume of saturated sucrose solution containing 0.001% bromophenol blue (Eastman Kodak) and run alongside OX174 HaeIII restriction fragments (BRL, MD) as marker DNA. Gels were run for 3 h at room temperature. Electrophoresis overnight or in the cold did not alter resolution.

Gels were stained by immersion in 5 $\mu g\,ml^{-1}$ ethidium bromide (Sigma) for 15 min, followed by a water wash for 15 min. DNA fluorescence was visualized using a 305 nm UV-light box, then photographed with a Polaroid instant camera. Photographs were scanned using a Quick Scan Jr. scanning densitometer (Helena Labs., Beaumont, TX) and migration of bands measured on the resultant traces. For each gel, calibration curves based on marker DNA migration were constructed and used to calculate the numbers of base pairs in the sample DNA bands.

**Figure 1** Electrophoresis of micrococcal nuclease-digested DNA from nuclei isolated from cultured L1210 cells (b), mouse bone marrow cells (c) and L1210 ascites cells (d). (a) = OX174 marker DNA. The arrow indicates the position of the nucleosomal monomer fraction. A further, irrelevant lane has been masked to fascilitate direct comparison of the important samples.

### Results

Figure 1 is an example of a gel on which DNA fragments from micrococcal nuclease-digested nuclei isolated from L1210 leukaemia (cultured and mouse ascites) and from mouse bone marrow cells were all run alongside OX174 HaeIII standard fragments. The enzyme digestion has produced banding patterns, representative of digestion of the chromatin into smaller molecular weights and indicating the presence of nucleosomal monomers (arrowed), dimers, trimers and larger oligomers. Differences are apparent in the distances to which the samples from different cellular origins have migrated. DNA from cultured L1210 cells (b) has migrated fastest and that from mouse bone marrow (c) the slowest. Quantitative values, in terms of length in base pairs (bp), were obtained by averaging the differences between the first (monomer) and next 4–6 bands. This method gives a more accurate measurement of linker length than using the values for the bands themselves as the enzyme trims the cut ends of the polynucleosomes during digestion (Noll & Kornberg, 1977). Table I shows the repeat lengths for the three cell types derived from 6 separate experiments, involving up to three measurements on each gel. It is apparent that L1210 ascites and cultured L1210 cells differ by the same amount as do bone marrow and L1210 ascites cells, that is by about 11 bp.

**Table I** DNA repeat lengths measured using electrophoresis of micrococcal nuclease-digested chromatin from mouse bone marrow and L1210 leukaemia cells. Values indicate numbers of base pairs Linker length = repeat length-146 bp

| | *Cell type* | | |
|---|---|---|---|
| *Expt. No* | *Bone marrow* | *L1210-Ascites* | *L1210-Culture* |
| 1 | 197.9 | 192.8 | 181.9 |
| 2 | 213.9 | 196.0 | 196.2 |
| 3 | 210.0 | 203.3 | 190.2 |
| 4 | 210.1 | 196.0 | 187.5 |
| 5 | 210.7 | 202.0 | 181.2 |
| 6 | 209.2 | 193.9 | 181.5 |
| Mean/sd | 208.6/5.0 | 197.3/3.9 | 186.4/5.5 |
| Linker length | 62.6 | 51.3 | 40.4 |

Data published previously (Green et al., 1982) showed the overall binding of CCNU and CLZ to bone marrow and L1210 ascites chromatin, in addition to the proportion of drug associated with micrococcal nuclease sensitive DNA. Using these values in conjunction with our own measurements of linker length it is possible to obtain estimates of drug binding to both linker and nucleosomal DNA, ($pmol\,mg^{-1}$ DNA). These values were then converted to estimates of alkylations/$10^5$ bp. These figures are shown in Table II and clearly show that more marrow-toxic CCNU binds to the nucleosomal DNA in both cell types and less to the linker DNA. CLZ, on the other hand, binds less to the nucleosomal DNA of bone marrow, but causes considerable alkylation of L1210 nucleosomal DNA. Furthermore, CLZ shows more alkylation of bone marrow, as compared with L1210, linker DNA.

**Table II** Estimated binding of CCNU and CLZ to linker and nucleosomal DNA in murine bone marrow and L1210 leukaemia cells from mouse ascites fluid. Numbers represent alkylations per $10^5$ base pairs

| *Drug* | *Cell type* | *Linker* | *Nucleosome* |
|---|---|---|---|
| CCNU | Bone marrow | 2 | 22 |
| | L1210 | 1 | 13 |
| CLZ | Bone marrow | 11 | 16 |
| | L1210 | 3 | 30 |

The banding patterns derived from electrophoresis of DNA from control and drug-treated, *in vitro* L1210 cells are shown in Figures 2 and 3. Treatment for 6 h with 1 mM CCNU (Figure 2) has no effect on micrococcal nuclease digestion, neither has treatment for 1 h with 4 mM CLZ or 1 mM L-PAM, (Figure 3). HN2, on the other hand at 1 mM for 1 h caused an increase in the proportion of mononucleosomal and smaller oligomer fractions, as well as a small reduction in the rate of migration, (Figure 3).

**Figure 2** Electrophoresis of micrococcal nuclease-digested DNA from nuclei isolated from cultured L1210 cells, untreated (b) or after incubation for 6 h with 1 mM CCNU (c). (a) = OX174 marker DNA. The arrow indicates the position of the nucleosomal monomer fraction.

### Discussion

Consistent and reproducible differences have been found in the repeat length of the DNA from bone marrow and L1210 leukaemia cells from mouse ascites. The bone marrow linker region (63 bp) was ~22% longer than that in the tumour (51 bp).

Drug binding data shown in Table II suggests that the toxicity of CLZ and, indeed, other chloroethylnitrosoureas, is related to the extent to which the drugs alkylate nucleosomal DNA. Reaction with linker DNA is probably less critical as evidenced by the binding of CLZ to bone marrow linker DNA. This correlates with reports of preferential repair of internucleosomal DNA (Bodell, 1977; Cleaver, 1977; Smerdon et al., 1979) which would render alkylation in this region less cytotoxic. It is possible that variation in cellular sensitivity to the different types of drug may be a result of differences in the ability of cells to repair drug-induced damage to linker versus nucleosomal DNA.

The observed repeat lengths of cultured L1210 cells and those grown *in vitro*, 186±5 and 197±4 bp respectively, are in approximate accordance with a report that cells in culture generally have a repeat length of less than 196 bp (Compton et al., 1976). It was also suggested that increased repeat length may correspond to increased cell growth and genetic activity. Murine bone marrow consists of a variety of cell types, varying in proliferative status from pluripotential stem cells to mature blood cells (Tavassoli & Yoffey, 1983) and it is estimated that only about 1 in 104 represent the colony forming units (Coggle & Gordon, 1975), the great majority being mature

**Figure 3** Electrophoresis of micrococcal nuclease-digested DNA from nuclei isolated from cultured L1210 cells, untreated (b) or after incubation for 1 hr with 4 mM CLZ (c), 1 mM HN2 (d) or 1 mM L-PAM (e). (a) = OX174 marker DNA. The arrow indicates the position of the nucleosomal monomer.

and hence non-dividing cells. Thus, not only have we observed a consistent and reproducible measurement of repeat length but we have seen a longer repeat length in a population containing a large proportion of non-dividing cells compared to both L1210 populations which were maintained in exponential growth.

It is also important to note that neither CCNU nor CLZ had any measurable effect on the digestion of L1210 DNA by micrococcal nuclease. This observation serves to validate previous studies which used micrococcal nuclease digestion of nitrosourea-treated chromatin (Tew *et al.*, 1978, Green *et al.*, 1982). The alteration of the digestion pattern seen after treatment with HN2 would advise caution if the site of binding of this drug were to be investigated by following the micrococcal nuclease-induced release of HN2 from treated chromatin.

In conclusion, a 22% longer DNA linker length in murine bone marrow cannot account quantitatively for the reported 2–4 fold increased binding of sugar nitrosoureas to linker DNA (Green *et al.*, 1982). The effective specificity of sugar nitrosoureas for tumour cells and their reduced myelotoxicity may lie in other structural or conformational differences in the chromatin and associated proteins in these two cell types.

This investigation was supported by NIH grant CA17583 and ACS grant CH-13f.

### References

BILLING, R.J. & BONNER, J. (1972). The structure of DNA as revealed by Deoxyribonuclease studies. *Biochem. Biophys. Acta*, **281**, 453.

BODELL, W.J. (1977). Non-uniform distribution of DNA repair in chromatin after treatment with methyl methanesulphonate. *Nucl. Acids Res.*, **4**, 3155.

CHAMBON, P. (1977). Summary: The molecular biology of the eukaryotic genome is coming of age. *Cold Spr. Harb. Symp. Quant. Biol.*, **42**, 1209.

CLEAVER, J.E. (1977). Nucleosomal structure controls rate of excision repair in DNA of human cells. *Nature*, **220**, 451.

COGGLE, J.E. & GORDON, M.Y. (1975). Quantitative measurements on the haematopoeitic system of three strains of mice. *Exp. Hematol.*, **3**, 181.

COMPTON, J.L., BELLARD, M. & CHAMBON, P. (1976). Biochemical evidence of variability in the DNA repeat length in the chromatin of higher eukaryotes. *Proc. Natl. Acad. Sci.*, **73**, 4382.

FELSENFELD, G. (1978). Chromatin. *Nature*, **271**, 115.

GOTTESFELD, J.M. & BUTLER, P.J.G. (1977). Structure of transcriptionally active chromatin subunits. *Nucl. Acids Res.*, **4**, 3155.

GREEN, D., TEW, K., HISAMATSU, T. & SCHEIN, P.S. (1982). Correlation of nitrosourea bone marrow toxicity with DNA alkylation and chromatin binding sites. *Biochem. Pharmacol.*, **31**, 1671.

GURLEY, L.R. & HARDIN, J.M. (1969). The metabolism of histone fractions II. Conservation and turnover of histone fractions in mammalian cells. *Arch. Biochem. Biophys.*, **130**, 1.

HANCOCK, R. (1969). Conservation of histones in chromatin during growth and mitosis *in vitro*. *J. Molec. Biol.*, **40**, 457.

HOTH, D.F., SCHEIN, P.S., WINOKUR, S. & 4 others (1980). A phase II study of chlorozotocin in metastatic malignant melanoma. *Cancer*, **46**, 1544.

NOLL, M. & KORNBERG, R.D. (1977). Action of micrococcal nuclease on chromatin and the location of histone $H_1$. *J. Molec. Biol.*, **109**, 393.

OSBAND, M., COHEN, H., CASSADY, J.R. & JAFFE, N. (1977). Severe and protracted bone marrow dysfunction following long-term therapy with methyl-CCNU. *Am. Soc. Clin. Oncol. Proc.*, **18**, 303.

PANASCI, L., GREEN, D., NAGOURNEY, R., FOX, P. & SCHEIN, P. (1977). A structure activity analysis of chemical and biological parameters of chloroethyl nitrosoureas in mice. *Cancer Res.*, **37**, 2615.

PANASCI, L.C., GREEN, D. & SCHEIN, P.C. (1979). Chlorozotocin, a mechanism of reduced bone marrow toxicity in mice. *J. Clin. Invest.*, **64**, 1103.

SMERDON, M.J., KASTOR, M.B. & LIEBERMAN, M.W. (1979). Distribution of repair-incorporated nucleotides and nucleosomal rearrangement in the chromatin of normal and Xeroderma pigmentosum human fibroblasts. *Biochem.*, **18**, 3732.

SOLLNER WEBB, B., MELCHIOR, W. & FELSENFELD, G. (1978). DNAse I, DNAse II and Staphylococcal nuclease cut at different, yet symmetrical, sites in the nucleosome core. *Cell*, **14**, 611.

TAVASSOLI, M. & YOFFEY, Y.M., (1983). Quantitation studies of bone marrow. in *Bone Marrow Structure and Function*, p. 125. Alan & Liss Inc. N.Y.

TEW, K.D., SCHEIN, P.S., LINDER, D.J., WANG, A.L. & SMULSON, M.E. (1980). Hydrocortisone modification of chromatin structure alters nitrosourea binding within the nucleus. *Cancer Res.*, **40**, 3697.

TEW, K.D., SUDHAKAR, S., SCHEIN, P.S. & SMULSON, M.E. (1978). Binding of chlorozotocin and 1-(2-chloroethyl)-3-cyclohexyl-1-nitrosourea to chromatin and nucleosomal fractions of HeLa cells. *Cancer Res.*, **38**, 3371.

THOMAS, J.O. & KORNBERG, R.T. (1975). An octamer of histones in chromatin and free in solution. *Proc. Natl Acad. Sci.*, **72**, 2626.

VU, V.T., TEW, K.D., AHLGREN, J.D. & 4 others (1983). Chloroethylnitrosourea interaction with chromatin: A potential mechanism for the bone marrow-sparing effect of the glucose analogs. *Cancer Treat. Symp.*, **1**, 37.

WASSERMAN, T.H., SLAVIC, M. & CARTER, S. (1975). Clinical comparisons of the nitrosoureas. *Cancer*, **36**, 1258.

WORCEL, A. & BENYAJATI C. (1977). Higher order coiling of DNA in chromatin. *Cell*, **12**, 83.